# Life Preserved

**Published:** 1858-10

**Authors:** 


### HALL'S JOURNAL OF HEALTH.

OUR LEGITIMATE SCOPE IS ALMOST BOUNDLESS: FOR WHATEVER BEGETS PLEASURABLE AND HARMLESS FEELINGS, PROMOTES HEALTH; AND WHATEVER INDUCES DISAGREEABLE SENSATIONS, ENGENDERS DISEASE.

*We aim to show how Disease may be avoided, and that it is best, when sickness comes, to take no Medicine without consulting an educated Physician.*

VOL. V.] OCTOBER, 1858. [No. 10.

### LIFE PRESERVED.

LESS than forty-eight hours ago, this September morning, six o'clock, in the year of grace, one thousand eight hundred and fifty-eight, we found ourselves in a spacious room, or rather suite of rooms, in Broadway, amid the beautiful in person, in paintings, and mechanics. The beauties on the wall, as well as those on the floor, might well have riveted the attention of younger men. There was blooming "seventeen"—the eye was bright and loving; there was intelligent sweetness in the face; the long curls of rich brown hair fell in profusion over the shoulders, reaching below the waist—while those which kissed the cheek in front, needed only actual life to make them happy. The maiden "*passée*" was there; the countenance was staid, serious, and reflecting, as if to say: "The last die for happiness has been thrown, and — lost! but I'll bear it." Maternity and widowhood were there; young men, married men, and men unblessed, were there. All were engaged. Intense interest pervaded every countenance. Fingers and feet were alike busy. Some were learners. Some had learned; and there was a pride of having accomplished something in all. But of the sights we saw that hour, there was one, which, for oddity and novelty, made upon our own mind an impression, so instantaneous and so deep, that it can never be effaced—just as deep, perhaps, as if the reader were this moment to see a man with a head, not on his shoulders, but at his ankles. In all thoughts of all past time, in all our dreamings, awake or asleep, such a thing, in present memory, had never occurred to our imagination; and before we knew it, our eyes were almost blinded with the tears of earnest straining. And what may seem stranger, and yet was true, wifey was along—she had seen it a hundred times; had handled it, used it, knew all about it; had never said a word respecting it to us, and seemed not to give it a thought. It was a needle—a sewing-needle! But the queerity of the thing was, the eye was at the pointed end. What a singular idea! and what a singular being he must have been, to have begotten such an idea from his brain; no doubt, the Messrs. FOWLER would find among the bumps of his head some one as unusual as the needle with its eye in the point. There he comes, now, up Broadway! The eye would single him out, instantly, from among the crowding thousands. The Kossuth hat—the rollicking gait—the head turning from shoulder to shoulder at every step, as of a man who did not under-estimate his importance. The long hair floats on either side on the shoulder, and is cultivated with care. The face is all covered with hair; but he calls his carriage, and away he dashes to his beautiful country-seat.

Less than twenty years ago, in Cornhill, Boston, there stood the rickety, deserted, old Museum Building; but soon, one of its craziest rooms was tenanted by a "poor genius." How often do they go together, both of them equally contemned by the purse-proud, successful few; it was dignified, in a short time, with the name of "The Philosophical Instrument Shop." On a certain day, a short, thick-set, ruddy, merry-faced, gray-eyed stranger visited the "Professor;" and for a whole hour they were engaged in quick, positive, earnest conversation, to which a youth of twenty-one, a kind of half-apprentice and half-journeyman, listened, as he worked at his vice, with eager attention; and concluded, then and there, in his own mind, that some things could be done as well as others. But he kept his counsel, "said nothing to no one," and thought, and thought, and thought—contriving and recontriving, the meanwhile, with the energy of a mind made up to high resolves. What that unfledged journeyman thought out, and hammered out, in that chicken-roost of a room, in the old Museum Building in Cornhill, Boston, in that three years' time, is saving human life, and will continue to save multitudes of the young, and innocent, and beautiful of our kind! Well may he, to-day, rollick along Broadway, in pleasant consciousness of his agency in alleviating human ills, with the reward of an annual income of over thirty thousand dollars, with no further trouble than in collecting it! That unfledged young journeyman, in the dirty old room in Cornhill, Boston, and the long-haired rollicker in Broadway, with his splendid equipage and princely country-seat, and the man who thought of putting a needle's eye in the wrong end, are one and the same person—that is, the FITCH and FULTON of the "*Sewing Machine.*"

Accomplishments elevate us. It is the "*fait accompli,*" the deed done, which makes one feel his manhood; and the prouder are we for the greater difficulties past. Those very difficulties, which awhile before were crushing the very life out of us, now overcome, lend wings to our upward flight, add sweetness to the cup, and perfume to the rose. So with Mr. HOWE. After three years of weary, wearing thought and toil, the first sewing-machine was put in operation; but nobody would pay any attention to it, and he took it to Europe. For long months of poverty, he hawked it about England and France for a purchaser; and at length, in destitution, sold it for a song, and returned home—not to give up, but to "try again," and for something better; and better did he succeed, for our people waked up to the importance of the invention. But just as a rich harvest was falling into his lap, wide-awake men pirated his patent, infringed upon his rights, and trumpeted their own bastard claims to the nation. They tricked, they contrived, they fabricated, and modified and altered, until nothing original was left in HOWE's sewing-machine; every thing was new, was actually improved upon. There was as much difference (and superiority) between HOWE's original sewing-machine and the best now in use, as there is between a common tea-kettle and a steam-engine—only one resemblance, only one thing the same (the steam), the eye in the wrong end of the needle! That, and that alone, with the aid of the legal acumen and eloquence of a BRADY, saved Mr. HOWE's patent! The eye in the wrong end of the needle has never been improved upon, and the presumption is, never will be. When Mr. HOWE gained his victory, he became generous; and calling all the sewing-machine manufacturers together at a threethousand-dollar dinner, at the most splendid hotel in NewYork, and at the moment when all were happy with wassail and with wine, said, in substance:

"GENTLEMEN: The essence of the invention is mine; the law has decreed it so. You thought your improvements entitled you to its free use. In making those improvements, you have labored hard and long; by that labor, have made it essentially a universally practicable instrument; and by your industry, enterprise, and liberality in advertising it into notice, you have hastened its introduction many years sooner than I should have done; because I had not the means, nor did I possess your tact—and you deserve the reward of the lion's share of the profits. Give me only a very small portion, according to your ability, for each machine you make, and henceforward we will pull together."

As a matter of historical interest, it may be well to add, and every intelligent reader will be gratified with the addition to an already long article, that while Mr. HOWE is the actual inventor of the sewing-machine, contrived it out wholly and originally as to himself, during 1842, 1843, and 1844, he was not the first inventor; that credit is due to WALTER HUNT, of this city, who, twenty-seven years ago, to wit, in 1831, or along there, made the first sewing-machine of modern times, but failed to push it into notice, and sold it to Mr. NESMITH, who laid it with some old lumber, where it remained safe, until it was burned up in one of our great fires; what the fire left, he picked out from the ashes, and saved with great care, as was evidenced by production in court, in Boston, on the occasion of the great trial. But Mr. HUNT'S testimony was ruled out in Boston, in consequence of his religious belief, or disbelief. So Mr. HOWE came off victorious—and rightly, too; for the covering up of a talent, or a light, or a useful invention, whether by overt design or simple neglect, is a crime against our common humanity—and in this case has met its reward. Honor and profit everywhere, say we, be to the men who show their light, that the toiling sailor at sea, in the midnight storms of human life, may see its beaming, take courage, and strike afresh for the haven of rest and of home.

The connection which this narration of actual facts has with a JOURNAL OF HEALTH, is simply this: that the general use of the sewing-machines which we have named, as to their effects in aiding over-tasked wives and women, in preventing a premature loss of eye-sight, and by their saving hours of weary labor every day, and thus allowing more time for refreshing sleep, for life-giving exercise, for domestic and social harmonies, is utterly beyond human calculation.

NEW YORK, September 9, 1858.

